# Quantifying Covid19-vaccine location strategies for Germany

**DOI:** 10.1186/s12913-021-06587-x

**Published:** 2021-08-07

**Authors:** Neele Leithäuser, Johanna Schneider, Sebastian Johann, Sven O. Krumke, Eva Schmidt, Manuel Streicher, Stefan Scholz

**Affiliations:** 1 Fraunhofer ITWM, Fraunhofer-Platz 1, Kaiserslautern, 67663 Germany; 2 TU Kaiserslautern, Gottlieb-Daimler-Straße, Kaiserslautern, 67663 Germany; 3 Robert Koch-Institut, Nordufer 20, Berlin, 13353 Germany

**Keywords:** Location planning, Covid-19, Vaccination planning

## Abstract

**Background:**

Vaccines are an important tool to limit the health and economic damage of the Covid-19 pandemic. Several vaccine candidates already provided promising effectiveness data, but it is crucial for an effective vaccination campaign that people are willing and able to get vaccinated as soon as possible. Taking Germany as an example, we provide insights of using a mathematical approach for the planning and location of vaccination sites to optimally administer vaccines against Covid-19.

**Methods:**

We used mathematical programming for computing an optimal selection of vaccination sites out of a given set (i.e., university hospitals, health department related locations and general practices). Different patient-to-facility assignments and doctor-to-facility assignments and different constraints on the number of vaccinees per site or maximum travel time are used.

**Results:**

In order to minimize the barriers for people to get vaccinated, i.e., limit the one-way travel journey (airline distance) by around 35 km for 75% of the population (with a maximum of 70 km), around 80 well-positioned facilities can be enough. If only the 38 university hospitals are being used, the 75% distance increases to around 50 km (with a maximum of 145 km). Using all 400 health departments or all 56 000 general practices can decrease the journey length significantly, but comes at the price of more required staff and possibly wastage of only partially used vaccine containers.

**Conclusions:**

In the case of free assignments, the number of required physicians can in most scenarios be limited to 2 000, which is also the minimum with our assumptions. However, when travel distances for the patients are to be minimized, capacities of the facilities must be respected, or administrative assignments are prespecified, an increased number of physicians is unavoidable.

## Introduction

In the expectation of an upcoming availability of vaccines against the SARS-CoV-2 virus, public health authorities already need to make appropriate preparations in order to utilize the available vaccine capacities from the very beginning. In politics, there are already numerous guidelines available on how to deal with such a large-scale vaccination which affects all countries simultaneously [1–5]. Besides from analyzing the actual number of people willing to get a vaccination [6, 7], one crucial decision, that researchers around the world currently study is the question of who to vaccinate first in the face of scarce resources (cf. [8, 9]). Our focus however lies on the logistic decision on where to administer the vaccine to the people. Aside from operational aspects, such as cooling the vaccination doses, availability of medical staff, or spacious waiting areas that allow social distancing, the people’s journey duration is an important factor to take into account, as it is assumed that it correlates with the willingness to be vaccinated [2, 10, 11].

The most convenient way of vaccine administration for the population would be a vaccination at the local physician as it is common for other vaccinations such as influenza or tetanus. However, given the expected shortness of doses in comparison to the demand in the initial phase of the vaccination campaign, such a decentralized setting on the one hand poses the risk of wastage due to fixed container sizes and non-adherence to vaccination recommendations on the other hand. The biggest disadvantages, however, are the technical aspects: In the most likely scenario of starting with mRNA-based vaccinations, the vaccine must be transported at about -70 °C and stored at -20 °C [12, 13], which may require cooling equipment or special logistics that cannot be provided by most physician practices.

In the absence of knowledge of the actual temperature and other technical requirements, the well-equipped university hospitals were therefore brought into discussion as possible vaccination sites. Since there are only 38 of them in Germany, it is obvious that the distances for the vast majority of citizens are significantly longer than in the general physician scenario. Bundling those to be vaccinated in a central location makes it easier to distribute the vaccinations only to the intended population cohorts; at the same time, however, many more people, especially from risk groups, meet in one place. In times of a high incidence, this increases the probability of a disease transmission during at the vaccination site running contrary to the intention of vaccination.

A compromise scenario is vaccinating the people in public health departments, which are also decentralised and could be equipped at least with less specialised cooling technology. Since on average several hundred people have to be vaccinated every day, it is still necessary to create a safe hygiene concept. Most likely, the vaccinations itself will not be performed in the health department building, but rather, e.g., in gymnasiums or other adjoining event halls, since the health departments themselves usually do not have the necessary space for waiting areas or the like. This interpretation is also valid in our greenfield studies, where arbitrary facilities in the area could be used.

This study aims to evaluate different locations scenarios for vaccination centers and different assignments of patients to these centers within a given time period. We do not aim to give detailed information on which patient is vaccinated at which center at what exact moment in time. Rather, we want to give information on how many patients from a fixed area, such as municipalities, are to be vaccinated at which center in a fixed time frame, such as a week. Thus our goal is to quantify the key indicators *number of required facilities*, *number of required physicians* and *patient’s travel distance* from their home to the assigned vaccination center. When it comes to optimization, we use a mathematical model and minimize some or all of the key indicators in a lexicographical order.

Another key indicator is the *vaccine wastage* which is implicitly considered in our method. Lacking detailed numbers of container sizes or vaccine perishableness, we do not model the wastage explicitly. However, we postulate that the weekly capacity of vaccine administration per physician should be aligned with the vaccine container sizes, i.e. a set of vaccine doses that has to be administered within a few days once opened. Then, minimizing the number of needed physicians directly implies the maximization of the physician’s occupancy rate and thus the minimization of vaccine wastage. Since we cannot provide the induced wastage ratio, we will give the utilization rates of doctors as a surrogate indicator.

The location scenarios studied in this paper are currently discussed by German decision makers. These are (I) vaccinating decentralized at local general practices, (II) vaccinating at public health departments throughout the country, or (III) vaccinating at few, but highly equipped university hospitals. Based on the health department locations of (II), which more or less correspond to counties, we also consider (IV) a greenfield approach, where we open only a subset of locations. However, the methods presented and used in this study can be adjusted to fit any given area and sets of possible vaccination centers.

This paper is structured as follows: In “Background and research design”, we first give some background information and explain our research design including considered parameters, objectives and assignment strategies. In “Methods”, we then explain the used methodology and give insights into the strength of the mathematical models. The main part of this contribution consists of a “Case study” section where we apply our methods to Germany. We start the case study with an data overview, present the four considered scenarios (I) to (IV) followed by the results and a short discussion on the sensitivities of our parameters and methods. We close the paper with an “Outlook” section on further aspects that yet have to be integrated into the model.

## Background and research design

At the base of this study we regard the situation that in each week we have a fixed amount of vaccinations that need to be distributed to the population. We assume that this number of vaccinations is the main bottleneck of the vaccination process, i.e. we regard the vaccination as a scarce resource and assume that there is no lack of people willing to be vaccinated. This is a realistic assumption for the first stage of the vaccination procedure in which the number of patients to be vaccinated greatly exceeds the number of available vaccinations. Thus, the main focus of our model is to ensure a complete distribution of all available vaccinations to the population in order to minimize the vaccination wastage. For later stages of the vaccination procedure, where more vaccinations are available and the bottleneck of the distribution problem moves towards finding people willing to be vaccinated, the proposed model cannot be applied directly. This situation is therefore not discussed in this contribution.

In order to get a sensible trade-off between complexity and significance, we decided upon using all (German) municipalities as basis regions for our evaluations and consider only linear distances between the municipality’s reference coordinate and a vaccination facility. The main variable in our model is the decision of which proportion of the municipality’s population should be sent to which vaccination facility. Depending on the specific scenario this variable often depends on the linear distance between the municipality and the vaccination facility, but can also be fixed beforehand by a given assignment. For each municipality, we calculate the part of its population to be vaccinated in the given time frame by scaling the total number of vaccination doses by the municipality’s population size. Here, it is also possible to consider, e.g., only elderly people or system-critical employees.

In this way, we ensure that the number of vaccinated citizens is distributed fairly across all regions. We also assume that there is a fixed amount of vaccine doses available per week and a maximum number of vaccinations each physician can administer in a week, see the subsection on “Data sources” for more details. In some scenarios we consider lower and upper bounds for the total number of patients that can be assigned to one vaccination center. These bounds can be used to avoid overloading a specific vaccination center as well as to ensure a fair distribution of the patients to the centers.

Beyond the above setting, the model is very versatile. In particular, it can handle a lot of different scenarios, i.e., we may choose different types of regions, vaccination centers, assignments between the regions and vaccination centers, etc. All in all, we use three decision variables in our model which can be identified with the following questions:

1. Which vaccination centers should be opened?
2. How many physicians are needed in each vaccination center?
3. Which citizens should be vaccinated in which vaccination center?

These are the main questions we ask for each scenario. We always aim to answer these questions (i.e. determine a solution for the decision variables) with specific goals in mind. Examples for these objectives are:

- Minimizing the number of open vaccination locations,
- minimizing the sum of the distances the patients have to travel for vaccination, or
- minimizing the number of physicians needed in total.

Here it is important that we can pursue not only one objective, but any lexicographic combination of these objectives. Other possible objectives may easily be added into the model. Minimizing the number of physicians per facility is not only important for logistic reasons (a physician may not be able to travel amongst different locations) and overhead costs (such as instruction courses, personal equipment, etc.), but can also directly be linked to the number of vaccine containers that will be opened and should not be wasted. We did not model the consideration of a certain container size explicitly, but it would be possible to do so.

We regard different patient to vaccination center strategies for all vaccination scenarios. We now describe the strategies used for this study. Note however that other strategies as well as combinations of objectives can easily be included into our model. We merely regard the most natural selection here. In all the strategies we assume that a set of potential vaccination centers is provided, we differ between different scenarios in this sense afterwards. *closest_station_greedy* In this perhaps most simple assignment strategy each patient is assigned to the closest vaccination center. In this case we open a possible center if at least one patient is assigned to it and the number of doctors assigned to the open centers is simply determined by dividing the number of patients by the weekly capacity of a doctor and rounding up. Note that this strategy works only for small vaccination container sizes, otherwise we might end up in more locations than available containers. However, the scenario serves as a reference for obtainable distances when the number of locations is not an issue. *closest_station_same_state* Similarly to the previous strategy, patients are assigned to the closest vaccination center within the same federal state. However, if no vaccination center within the same state exists, patients are assigned to the closest center overall. This analysis is reasonable, since the vaccine doses will probably be distributed among the states according to their population sizes. For the respective state governments, it is an undesired effect that their assigned vaccinations will be served to people from adjacent states. On the other hand, the convenience for their respective citizens will also be of concern. *responsible_station* In some scenarios a given region is assigned to a certain vaccination center due to administrative reasons. For example, each municipality in Germany has an assigned health department. In this strategy we assume that all patients are vaccinated at the corresponding responsible station. *free_assignment* Here, we assume that a general planner may decide which portion of the population is vaccinated at which facility. This could for example be realized by sending out vaccination notification to the population by the government. Of all feasible assignments, this strategy chooses one with as few as possible open vaccination centers, as few as possible doctors, and a minimum overall travel distance for patients, where the objectives are prioritized in the given order. An assignment is feasible if no patient has to travel further than a given distance bound to the assigned vaccination center and the number of patients assigned to each vaccination center is within the provided lower and upper capacity bounds. With this strategy, overhead costs for physicians is minimized as we minimize the number of needed doctors. *free_assignment_only_distance*: Similarly to the previous strategy, we choose an assignment out of all feasible assignments that minimizes the total travel distance of patients and the number of doctors needed to perform the vaccinations. Again the objectives are prioritized in the given order. Although technically a general planner is necessary to enforce this strategy, it can also be regarded as a possible strategy where patients choose where they are vaccinated since the first objective optimizes the travel distance. Therefore we use this strategy as a replacement of *closest_station_greedy*, whenever capacities are assumed as in this case *closest_station_greedy* most likely leads to infeasible solutions.

Before we apply these strategies in our case study, we present our methods including the mathematical model.

## Methods

The task of realizing the assignment strategies described in the previous section can be seen as an assignment problem. To deal with this problem we use two different algorithmic approaches. The first approach is based on a simple and fast greedy algorithm. More precisely, we determine for every region the vaccination station where the citizens of this region are vaccinated. The exact method how these stations are determined depends on which scenario we are interested in, i.e. the nearest station, the nearest station in the same state or the responsible station. Afterwards, we compute the number of people that are vaccinated in each station and determine the number of necessary physicians. In this way we solve the scenarios *closest_station_greedy*, *closet_station_same_state* and *responsible_station*.

The second approach is more involved and uses ideas from [14]. To compute assignment strategies for the scenarios *free_assignment* and *free_assignment_ only_distance* we solve an integer program. Integer programming deals with the optimization of (linear) objective functions over a set of possible integral solutions constrained by linear equations and inequalities. For a general introduction and overview we refer to [15]. While linearity may seem like a severe restriction, together with the integrality conditions integer programming allows to model complex correlations. Integer programming is NP-complete, which means that in general there is no polynomial time algorithm, which solves all instances to optimality, unless P =NP. Nevertheless, exponential time algorithms are available. Thus, to solve the integer program corresponding to our model we use Gurobi Optimizer, cf. [16]. Providing a detailed description on how integer linear programs may be solved is beyond the scope of this paper. Thus, again, for more details we refer to [15]. A comparative analysis of commercial and open-source solvers can be found in [17].

As described above we use different objects for our model. To keep the overview we summarize all these objects here.

- **The set J of regions:** A set of areas where the citizens to be vaccinated live (e.g., municipalities).
- **The set I of vaccination stations:** A set of possible vaccination centers (e.g., general practices, health departments, university hospitals).
- **The neighborhood N(j)** of the region **j∈J**: Possible vaccination centers that are responsible for the region *j* (e.g., determined by distance or predefined assignment).
- **The neighborhood N(i)** of the station **i∈I**: Regions from which citizens can be vaccinated in vaccination center *i* (e.g., determined by distance or a predefined assignment).
- **Number of citizens*d***_***j***_** to be vaccinated in region j** (e.g., $d_{j}=\frac {\text {total available vaccines}}{\text {population in Germany}}\times \text {population in region }j$)
- **Maximal capacity of vaccinations** **ca*p***_***i***_** in station** **i∈I** (e.g., can be set to infinity or another predefined value)
- **Lower Bound of vaccinations l*b***_***i***_** in station** **i∈I** **if station*i* is opened as a vaccination center** (e.g., can be set to 0 or another predefined value)
- **Maximal number of vaccinations b that can be carried out by a physician** (e.g., can be set to 250)

The remarks in the brackets are suggestions for these values and we mainly deal with these stated possibilities. Observe that these values always refer to the time frame that is fixed, in our case these values correspond to a 5-day week with 8 working hours each. However, if the appropriate data is available, these values can be replaced at will. This shows that the model is very universal and adaptable for different scenarios.

In the integer program we use three different variables. In the following we describe their purpose:

- *x*_*i*_∈{0,1} for *i*∈*I*: For a vaccination center *i*∈*I* the variable *x*_*i*_ states in a solution whether the vaccination center *i* is opened (*x*_*i*_=1) or closed (*x*_*i*_=0).
- $\mathbf {y_{i}}\in \mathbb {N}$ for *i*∈*I*: For a vaccination center *i*∈*I* the variable *y*_*i*_ states in a solution the number of physicians needed in vaccination center *i*.
- $\mathbf {z_{i,j}}\in \mathbb {N}$ for *i*∈*I* and *j*∈*J*: For a vaccination center *i*∈*I* and a region *j*∈*J* the variable *z*_*i*,*j*_ states in a solution how many citizens from region *j* are vaccinated in vaccination center *i*.

Thus, in a solution (*x*,*y*,*z*) variable *x* yields an answer to question 1, variable *y* gives an answer to question 2 and variable *z* implies an answer to question 3. We are now ready to present the integer program, where we abbreviate the objective function with a placeholder function *f*(*x*,*y*,*z*)=*a*^*T*^*x*+*b*^*T*^*y*+*c*^*T*^*z*:

1a $$\begin{array}{*{20}l}\text{(IP~1)} \quad \min\limits_{x,\,y,\,z} \quad f(x,y,z)& \end{array} $$

1b $$\begin{array}{*{20}l}  & \mathrm{s.t.} \quad\quad\quad\!\!\! {b\cdot y_{i}}\ {\leq \operatorname{cap}_{i}\cdot x_{i} } \quad\, {\forall i\in I} \end{array} $$

1c $$\begin{array}{*{20}l}  {b\cdot y_{i}}\ &{\geq \operatorname{lb}_{i}\cdot x_{i} } \quad\ \ \,\, {\forall i\in I} \end{array} $$

1d $$\begin{array}{*{20}l}  {\sum_{i\in N(j)}z_{i,j}}\ &{= d_{j} }\quad \quad \quad\, {\forall j\in J} \end{array} $$

1e $$\begin{array}{*{20}l}  {\sum_{j\in N(i)}z_{i,j}}\ &{\leq b\cdot y_{i} }\quad \quad\!\, {\forall i\in I} \end{array} $$

1f $$\begin{array}{*{20}l}  {x_{i}}\ &{\in \{0,1\} }\quad \quad {\forall i\in I} \end{array} $$

1g $$\begin{array}{*{20}l}  {y_{i}}\ &{\in \mathbb{N} }\quad \quad \quad\ \, {\forall i\in I} \end{array} $$

1h $$\begin{array}{*{20}l} {z_{i,j}}\ &{\in \mathbb{N} }\quad \quad \quad\ \, {\forall i\in I, \forall j\in J} \end{array} $$

In (IP 1) constraint (1b) ensures that if a vaccination center is open (i.e., *x*_*i*_=1), then there are at most as many possible vaccinations by the physicians as the capacity cap*i* of the vaccination center *i* allows. In particular, this bounds the number of physicians in each vaccination center. Constraint (1c) makes sure that if a vaccination center *i*∈*I* is open, then at least lb*i* vaccinations are carried out in *i*. In constraint (1d), we guarantee that, in each region *j*, exactly *d*_*j*_ citizens are being vaccinated. Note that, in some scenarios, these citizens are vaccinated in different facilities. Constraint (1e) ensures that all vaccinations in vaccination center *i* can be performed by the physicians assigned to *i*. The model is a variant of a *facility location problem*. For a general overview on facility location we refer to [18, 19]. Another facility location model used to select pharmacies for Covid19-testing is given in [20]. For a more recent survey on health care related facility location problems we refer to [21].

The model above is quite universal, different objective functions allow to focus on various goals. We have implemented various objective functions and give a short overview in the following:

- **Minimizing the number of open vaccination stations:**$f(x,y,z)=\sum _{i\in I}x_{i}$ (*y* and *z* are not used).
- **Minimizing the number of needed physicians:**$f(x,y,z)=\sum _{i\in I}y_{i}$.
- **Minimize the sum of the travel distances of the patients**For *i*∈*I* and *j*∈*J* compute distance dist(*i*,*j*) from *i* to *j* and set $f(x,y,z)=\sum _{i\in I,j\in J}z_{i,j}\cdot \operatorname {dist}(i,j)$.

It is important to note that we can optimize not only a single one of these objective functions, but also multiple of them in any lexicographical combination. If, for example, our given combination is (a) and (b), this means that when optimizing the second objective (b) we only consider optimal solutions of objective (a) as possible solutions. Therefore, we obtain different assignment strategies for the doctors and information which patient should be vaccinated at which vaccination center in our scenarios.

For the assignment strategy *free_assignment* we now solve the provided integer program with the objectives (a), (b) and (c) in the provided order. For the assignment strategy *free_assignment_only_distance* on the other hand we solve the integer program with the objectives (c) and (b) in the provided order.

All code needed for the computations was written in Python 3. The computations of the first algorithmic approach were completed on a standard home computer. The solution process of the integer program was completed on a machine with 32 cores, each of which running at 3.2 Ghz, where the machine has a total of 94.3 GB RAM main memory.

## Case study

Before we present the four different location scenarios considered in this case study, we give some information about our chosen parameters and used data.

### Data sources

In our studies we consider a weekly cycle. Our personal correspondence with Stefan Scholz from the RKI has shown that their current estimate is to have around 500 000 vaccine doses available per week. They further assume that a doctor needs about 10 minutes on average for a vaccination and can therefore vaccinate 250 patients in a 5-day week of 8 hours each. The theoretical minimum is therefore a total of 500 000/250=2 000 physicians. Further, we received data about university hospitals and health departments from the RKI. We have obtained the population data of the municipalities in Germany from the federal statistical office (*Statistisches Bundesamt*) in Germany, cf. [22]. The data concerning general practices has been collected by Stefan Scholz and Katharina Schmidt in 2013 using publicly available data and can be found on the web, cf. [23]. Of course, this number has changed in the meantime, but the available data is still useful for a first guess of the distribution of general practices in Germany. If we obtain newer data sets, we can easily add them and adjust our results. Further, data on the age structures in Germany is provided on a website of the *Bundesamt für Bauwesen und Raumordnung*, cf. [24]. Since our main concern is to compare the resulting proportions of the key figures, the different ages of the data are not problematic.

According to the current political discussions and our personal communication with the RKI, the doses shall be distributed among the states according to their population size as shown in Table 1 [1].

**Table 1:** Distribution of weekly vaccinations per state

	State	Abbreviation	Population	Vaccinations
1	Baden-Wuerttemberg	BW	11 069 533	66 670
2	Bavaria	BY	13 076 721	78 761
3	Berlin*	BE	3 644 826	21 952
4	Brandenburg	BB	2 511 917	15 134
5	Bremen*	HB	682 986	4 113
6	Hamburg*	HH	1 841 179	11 089
7	Hesse	HE	6 265 809	37 734
8	Mecklenburg-Vorpommern	MV	1 609 675	9 697
9	Lower Saxony	NI	7 982 448	48 071
10	North Rhine - Westphalia	NW	17 932 651	108 005
11	Rhineland Palatinate	RP	4 084 844	24 591
12	Saarland	SL	990 509	5 965
13	Saxony	SN	4 077 937	24 569
14	Saxony-Anhalt	ST	2 208 321	13 297
15	Schleswig-Holstein	SH	2 895 229	17 440
16	Thuringia	TH	2 143 145	12 912
	**Total**		**83 017 730**	**500 000**

^*^city states with BE and HH consisting of only one and HB of two municipalities

An overview of the sources of the data is provided in Table 2.

**Table 2:** Data sources

Data Type	Data Source
Population data	Statistisches Bundesamt Deutschland [22]
General practices	ZEIT ONLINE [23]
University Hospitals	Personal correspondence with the RKI [25]
Health Departments	Personal correspondence with the RKI [25]
Age structure	Bundesamt für Bauwesen und Raumordnung[24]
Vaccine distribution	Bundesgesundheitsministerium [1]

We also consider the whole German population as the reference population that should be vaccinated. In “Sensitivity analysis”, we discuss the sensitivity of the results to the weekly vaccination capacity, the weekly doses of vaccine available and partial populations.

### Scenarios

In our personal correspondence with the RKI [25], we identified four different vaccination scenarios for Germany, namely a decentralized vaccination in general practices (I), a centralized vaccination at university hospitals (III), an intermediate scenario with public health departments (II) and a greenfield planning scenario (IV). For all scenarios, we first give a brief overview in the following. We then state the results in “Results”.

#### Vaccination in general practices (I)

The general medical care is one of the cores of the German health care system. As a rule, every German citizen has compulsory health insurance and pays a health insurance contribution, which depends on a percentage of the income from employment. In return, everyone is entitled to free medical services at any time and can consult any doctor who is authorized to treat them under the statutory health insurance system. In our model we consider about 56 000 general practitioners in Germany, cf. [23], where the practices are distributed in such a way that one can usually reach the nearest one within around 5 km, cf. [26]. In addition to the easy accessibility of the general practitioners, they also carry out general vaccinations. Therefore, general practices are a suitable and reasonable choice for vaccination centers and the most convenient solution for the patients. We model multiple licensed doctors within a joint practice or within the same building as separate facilities with separate capacities.

In the case of general practitioners providing the vaccinations we consider the assignment strategies *free_assignment* and *free_assignment_only_distance* with a maximum travel distance of 50 km. Note that the scenario of each person choosing her closest physician would lead to overcrowded practices at some places, where more than 250 patients would choose the same doctor. However, the resulting extra travel distance in comparison to *free_assignment_only_distance* is negligible.

#### Vaccination at public health departments (II)

Health departments are the local authorities that are part of the German public health service. They are responsible for the execution of the medical tasks of the health administration. In Germany there are almost 400 health departments and their duties are defined by federal laws, federal state laws and federal state regulations. Each municipality is usually assigned to exactly one health department, which in turn is assigned to exactly one federal state. Exceptions are the city states Berlin and Hamburg, which are modeled as big municipalities due to the used input data. However, due to their population size, they have in fact several health departments and it would thus be reasonable to subdivide them into city districts. Vice versa, most of the authorities are responsible for exactly one county. Some health departments are responsible for two counties, usually a city county and its surrounding county.

If a municipality is in a certain federal state, then it is assigned to a health department in the same federal state. The federal states are partly autonomous and hence take over the tasks of the health services for their citizens.

Health departments are already responsible for many health-related tasks and are therefore an obvious choice for vaccination centers. For health departments we regard the two assignment strategies *responsible_station* and *closest_station_greedy*. The first seems intuitive as the federal structure of Germany suggests that each resident of a municipality is vaccinated at the responsible health department. In the second approach, for comparison, we neglect this federal structure and instead assign each municipality to the closest health department. Due to a lack of data, we do not consider capacities of the individual health departments in terms of patients or doctors yet. However, if provided, this can easily be integrated into our model.

#### Vaccination at university hospitals (III)

Another possibility that can be considered for vaccination centers are hospitals. Here we consider university hospitals in particular as they are the largest and most modernly equipped hospitals. There are 38 university hospitals spread all over Germany which are mainly located in large cities. The connection between rural areas and the big cities is mostly well maintained, but in some cases there are municipalities more than 100 km away from the closest university hospital.

However, university hospitals fulfill the technical requirements for the storage and cooling of the vaccine in any case and are therefore considered as possible vaccination centers. With these assumptions we consider the three assignment strategies *closest_station_greedy*, *closest_station_same_state*, and *free_ assignment_only_distance*. For the latter, the lower and upper bounds on the university hospitals are set proportional to the sizes of the university hospitals, or more precisely, to the number of outpatients they treat. We set the bounds to ±20% of this value. Thus, if the hospital opens as a vaccination center, the number of assigned citizens has to respect these bounds. The analysis of the level of federal states is of importance here, since the university hospitals are not evenly spread in Germany and in part, they lie on the border with other federal states.

#### Greenfield planning (IV)

At last, we study a free planning approach, where the number of facilities is dependent on a defined maximal acceptable radius.

For the sake of simplicity we use the coordinates of the health departments as potential set of vaccination facilities. We also assume that their geographical distribution roughly reflects Germany’s population density. We interpret those as arbitrary facilities and in particular, we do not open all potential facilities for vaccination.

In this scenario we want to investigate the minimum number of needed vaccination facilities depending on a given maximum travel distance. Therefore our primary goal is to minimize the number of open locations, the secondary goal is to minimize the needed physicians and last, the actual travel distances is minimized. Thus in this scenario, we only consider the strategy *free_assignment* with different distance bounds.

Observe that there are some (rural) municipalities which are not within a given distance of a potential facility. These municipalities are assigned to their closest vaccination facility which therefore has to be open.

### Results

In the following, we analyze each scenario individually according to the linear distance between the municipality centers and the assigned vaccination stations, the number of physicians and locations and their median utilization rates. We further give more detailed insights that are relevant to the specific scenarios. In particular, we visualize all the selected locations on a map. Subsequently, we give comparative results for all scenarios.

For various analyses throughout this section, we use boxplots as a mean of visualizing distribution information. We orient ourselves by the default setting [27] of refined boxplots, i.e. drawing the boxes as the lower and upper quantile around the median and drawing whiskers that specify 1.5 times the IQR (interquartile range) [28]. Data outside this range is identified as outliers and drawn as small diamonds.

First we look at vaccinations at general practices.

#### Vaccination in general practices (I)

The general results for this scenario are given in Table 3.

**Table 3:** Results for general practices scenario

	Strategy	Med. Distance	#Locations	#Physicians	Med. Utilisation
	*free_assignment*	0.8 km	2000	2000	100%
	*free_assignment_only_distance*	0.5 km	7021	7021	14%

**Fig. 1:**
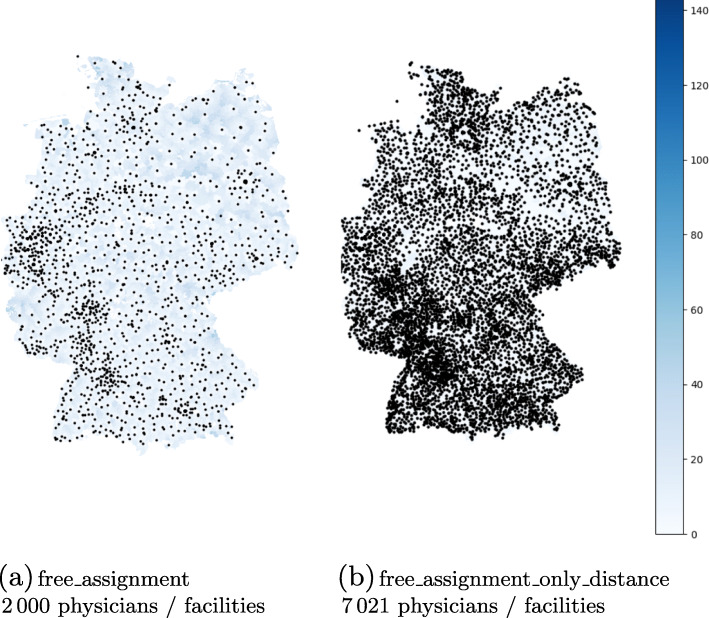
Visualization of the position of opened general practices. The municipalities are colored with respect to their airline distance in kilometers to their assigned physician. As shown in Fig. 2b, almost all municipalities are assigned to a practice closer than 10 km in the right figure and would be colored very lightly

**Fig. 2:**
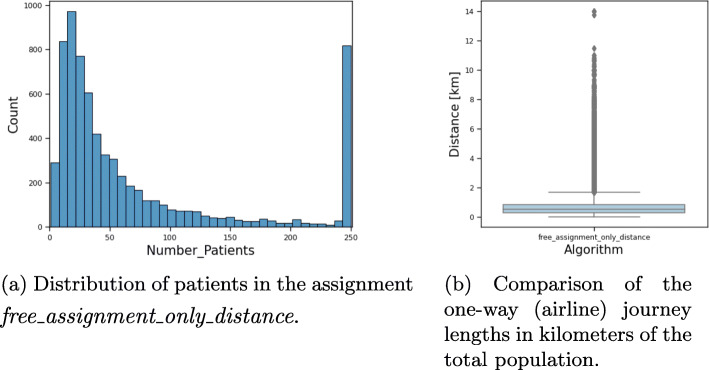
Distribution details for assignment ii in the general practices scenario

For reasons of space, we abbreviate the “General Practices” scenario in the evaluation charts with “GP”. Due to the high density of physicians in Germany, mapping all locations would not be of any help. The map in Fig. 1 however shows a reduced subset of the resulting open practices for the above assignment variants. For the sake of consistency with other maps later in this paper, the municipalities are also colored according to their median distance with respect to their assigned physicians. As we are limiting our analysis to the linear distances between the facilities location and the center point of a municipality, the detailed analysis of the distances is not very precise. It can however, easily be acknowledged that the vast majority of patients will be assigned to a rather close facility in both scenarios. By design, the distances are even smaller in assignment ii, where this is our primary goal, although the presented solution is not proven to be optimal, since the running time exceeded the preset time constraints. The distribution of distances are visualized in Fig. 2b. The median distance is very small in both assignments. Even in the *free_assignment* the assigned physician is closer than 20 km for over 97.5% of the population.

The main result in this scenario is that around 7 000 open practices out of 56 000 possible ones are enough to reach the best possible distance for the total population. Another important hint to deciders is that opening more than 2 000 locations automatically leads to a guaranteed wastage of vaccine doses if the number of shots per package is calibrated to the vaccination capacity of one full-time physician. This effect is visualized in Fig. 2a. Since the reduction of open locations is here only a secondary goal, almost all physicians will not work to capacity, which may result in vaccine wastage. In the *free_assignment*, all physicians vaccinate exactly 250 patients.

#### Vaccination at public health departments (II)

The general results for this scenario are given in Table 4.

**Table 4:** Results for health department scenario

	Strategy	Med. Distance	#Locations	#Physicians	Med. Utilisation
	*closest_station_greedy*	6.1 km	389	2191	52%
	*responsible_station*	7.6 km	375	2193	45%

Both assignments i and ii are fixed and only the number of necessary doctors and the distribution of distances need to be calculated.

**Fig. 3:**
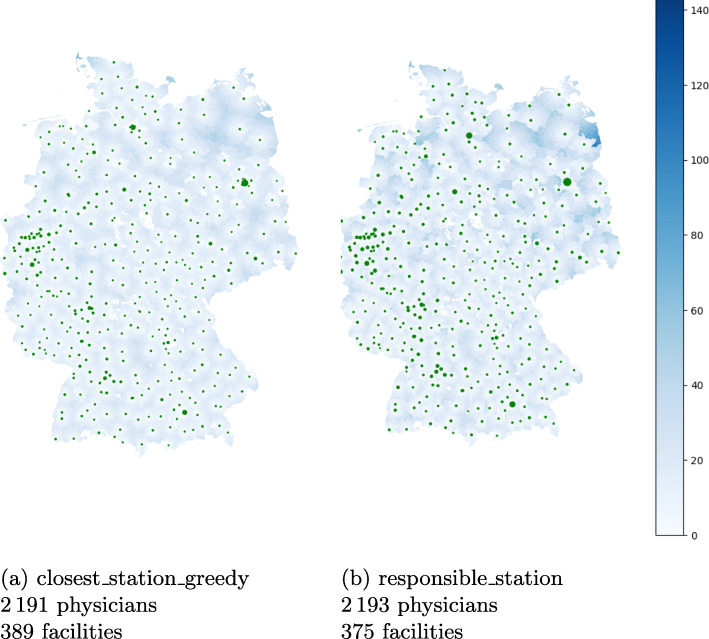
Visualization of the distance per municipality in kilometer airline for assignments i and ii in the health department scenario. Due to rounding issues, some very small municipalities are not assigned to any facility center and are kept white. The size of the green markers are proportional to the number of assigned patients

Figure 3 shows the location of all health departments on a map. As previously, each municipality is colored according to its travel distance to the assigned health department. Note that here, for each municipality, its assigned health department is unique for both assignments. Comparing the two assignments *responsible_station* (Fig. 3b) and *closest_station_greedy* (Fig. 3a) shows that the distances are quite similar in the southern and western part of Germany, but increase in the northern and eastern part. Especially the distances for the patients in the southeastern part of Mecklenburg-Vorpommern increase significantly as their reference point is much closer to a health department of another county than to their responsible one. This is also shown in Fig. 5a where we plotted the travel distances for both assignments for each federal state separately. For city states, some stations are not being assigned to any municipality, since no capacities are enforced. The differences in open facilities stems from the city states that have multiple departments, but only one is responsible for the whole city in our definition.

**Fig. 4:**
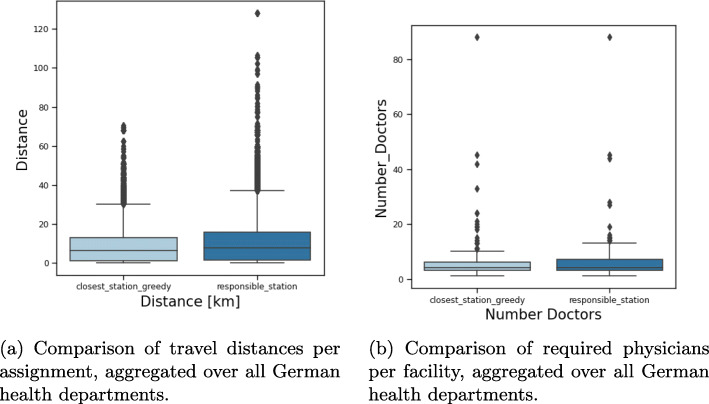
Comparison of assignments i and ii in the health department scenario aggregated over Germany

Figure 4a shows the increase in distances aggregated for Germany. The maximal distance of 128 km is attained for the remote island Helgoland. However, other large distances stem also from mainland Mecklenburg-Vorpommern.

The marker size in Fig. 3 refers to the number of physicians needed at the facility to vaccine the assigned patients. The maximal number of physicians per facility is attained in Berlin with 88 physicians. Since we do not subdivide big cities with several health departments into several districts, all patients of those cities are assigned to a single health department. Therefore, in those health departments as in Berlin and Hamburg, many physicians are required whereas in practice, these physicians would be spread over several health departments. This also explains the outliers in Fig. 4b where the distribution of the number of physicians per health department is shown.

**Fig. 5:**
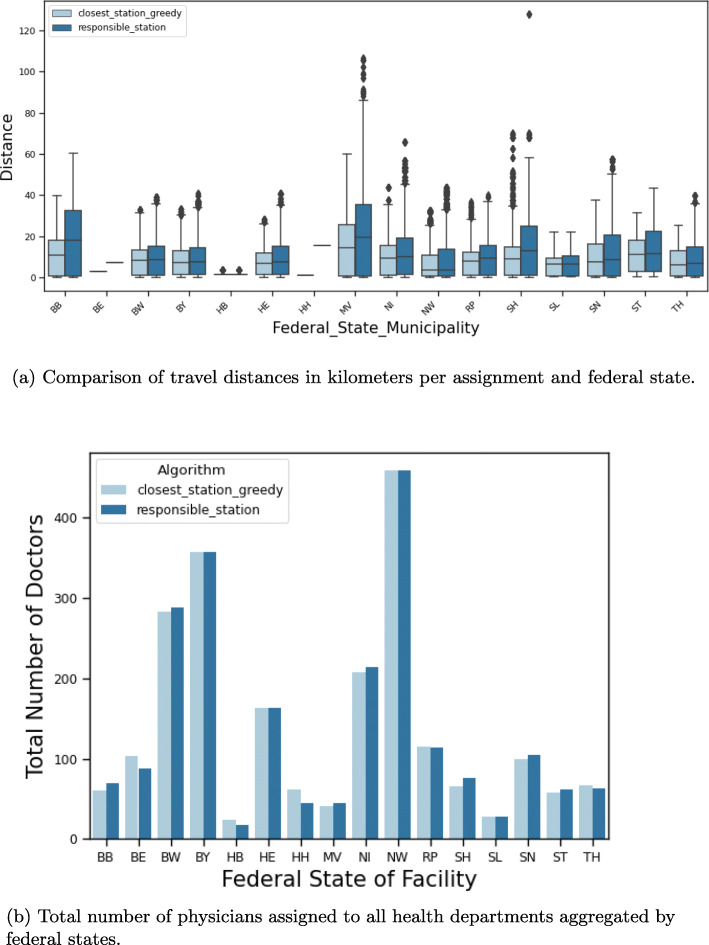
Comparison of assignments i and ii in the health department scenario

The total number of physicians for the considered assignments is 2 191 and 2 193, respectively. Figures 4 and 5 shows in more detail how the physicians are distributed among Germany (4b) per facility and accumulated per federal state (5b). The city states naturally have the least distances. The sparsely populated Mecklenburg-Vorpommern has the most disadvantages when sticking to the administrative assignment. As expected the federal states with the most inhabitants also require the most physicians. It varies between 24, respectively 17, in Bremen and 459, respectively 459, in North Rhine-Westphalia, but the difference between the two assignments is negligible. Also the number of required physicians per health department is similar for the two assignments as shown in Fig. 4b. The number of patients that are being assigned to a facility per week follows in principle the same distribution and varies from 30 to 22 200 with a median of around 1 000, i.e. 200 patients per weekday. Ignoring the outliers, 80% of all facilities would roughly face 500 to 2 000 patients per week, which translates into 2 to 4 required physicians in our setting.

#### Vaccination at university hospitals (III)

The general results for this scenario are given in Table 5.

**Table 5:** Results for university hospital scenario

	Strategy	Med. Distance	#Locations	#Physicians	Med. Utilisation
	*closest_station_greedy*	28.8 km	38	2017	55%
	*closest_station_same_state*	31.2 km	38	2018	55%
	*free_assignment_only_distance*	29.3 km	37	2010	96%

There are 38 university hospitals in Germany, spread over 14 of 16 federal states. Brandenburg and Bremen do not host a university hospital. Therefore, their citizens will be assigned to another university hospital within a 150 km radius. Note that there are two university hospitals in Munich very close by. One of them was not opened in assignment iii in order to save physicians.

**Fig. 6:**
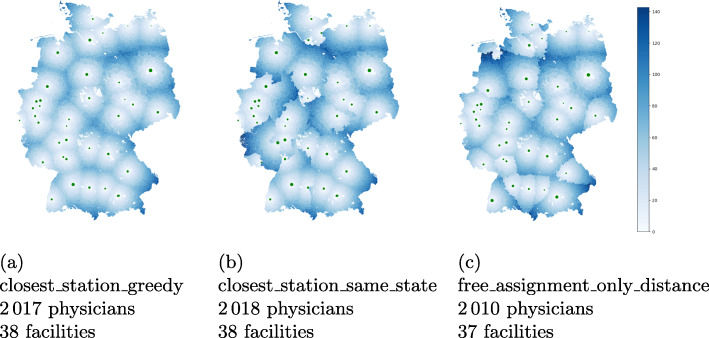
Visualization of the median distance per municipality in kilometer airline for the specified assignments in the university hospital scenario. Due to rounding issues, some very small municipalities are not assigned to any facility center and are kept white. The marker sizes are proportional to the number of assigned patients

**Fig. 7:**
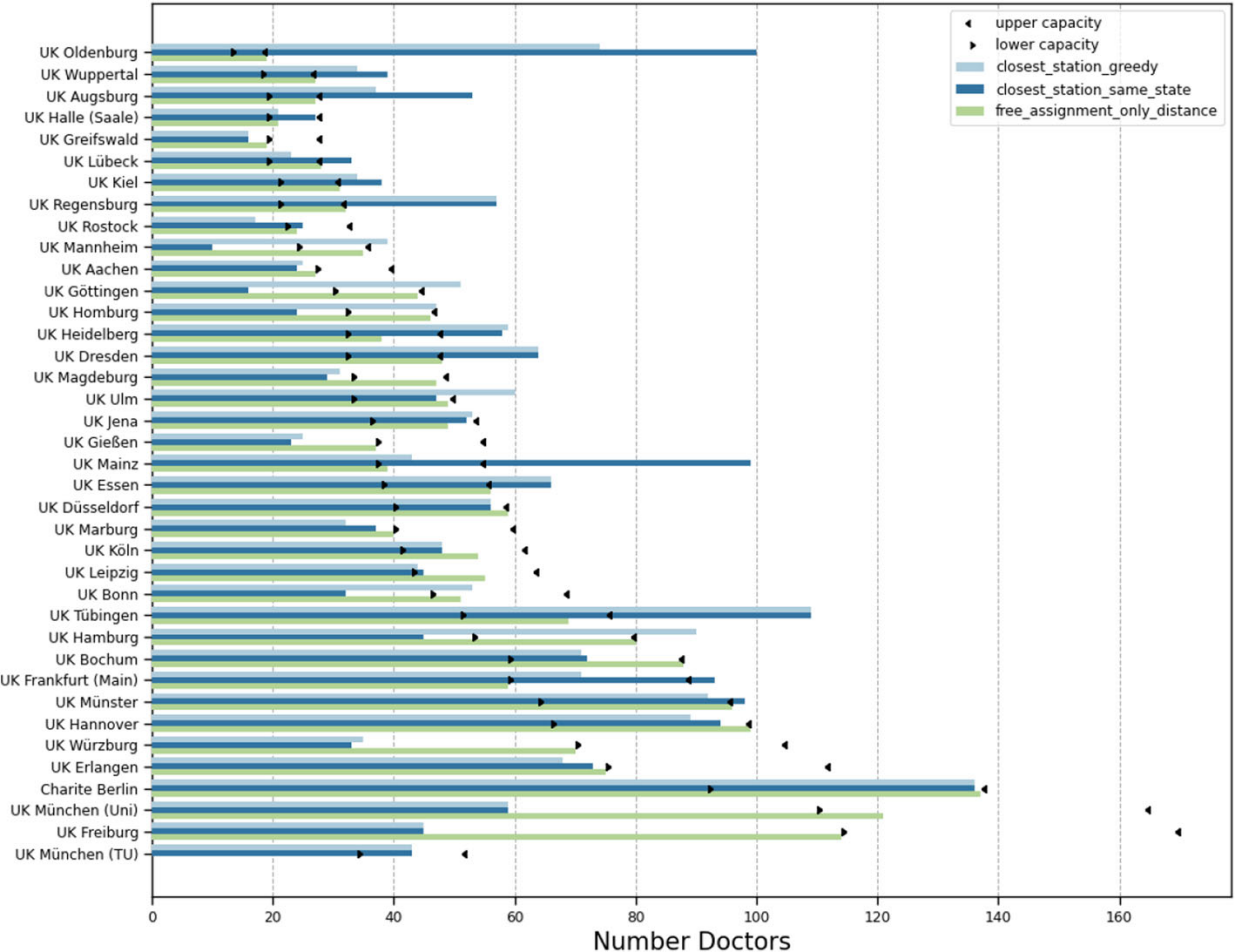
Number of vaccinating physicians assigned to each university hospital for i in comparison to ii and iii. Only for the latter, the depicted capacity bounds had been taken into account in the model

Figure 6 shows the locations of the hospitals on a map. Additionally, each municipality is colored according to its median (w.r.t. the population) travel distance to all assigned university hospitals. Note that only in Fig. 6c, a municipality can be assigned to multiple vaccination facilities. The marker sizes hint at the required number of physicians, which can be seen in more detail in Fig. 7.

For assignment i, the number of physicians per hospital vary between 16 doctors (Greifswald) and 136 doctors (Berlin). In total 2 017 doctors are required, each of whom can vaccinate up to 250 patients per week. For comparison, 2 018 doctors are needed for assignment ii and 2 010 doctors for assignment iii with capacity constraints. Note that the latter one is not necessarily solved to optimality. Our model can also compute feasible assignments with exactly 2 000 physicians, by slightly increasing the travel distances and deviate from a fixed assignment and e.g. distribute people from the same municipality to different facilities. Since the gap is rather insignificant and the distances do not alter visually, we omit this scenario for the sake of clarity. If we omit the capacities, we also can compute a solution with 2 000 physicians.

For assignment i (Fig. 6a), we see a circular increase in the distances around the university hospitals since everyone goes to the closest location. On the map in the middle (Fig. 6b) the borders of the German federal states are clearly visible. It shows some substantial distance increases due to the constraint that vaccination is only allowed in the own federal state. This is especially the case for some regions in Rhineland-Palatinate, North Rhine-Westphalia and Lower Saxony. Further, the map on the right (Fig. 6c) illustrates the increase of travel distance due to capacity constraints of the hospitals. The most noticeable difference can be seen around Oldenburg (Lower Saxony) in the north since the hospital capacity is much lower than the number of patients around. This is confirmed in Fig. 7 where the difference between the number of physicians (which are proportional to the number of patients) in assignment i to iii are plotted. Beside the university hospital in Oldenburg, the number of patients in other hospitals such as Regensburg (Bavaria) and Tübingen (Baden-Wuerttemberg) is also reduced due to the capacity limit. In contrast to that, some hospitals such as Freiburg (Baden-Wuerttemberg) and Würzburg (Bavaria) were allocated more patients because of a lower barrier.

**Fig. 8:**
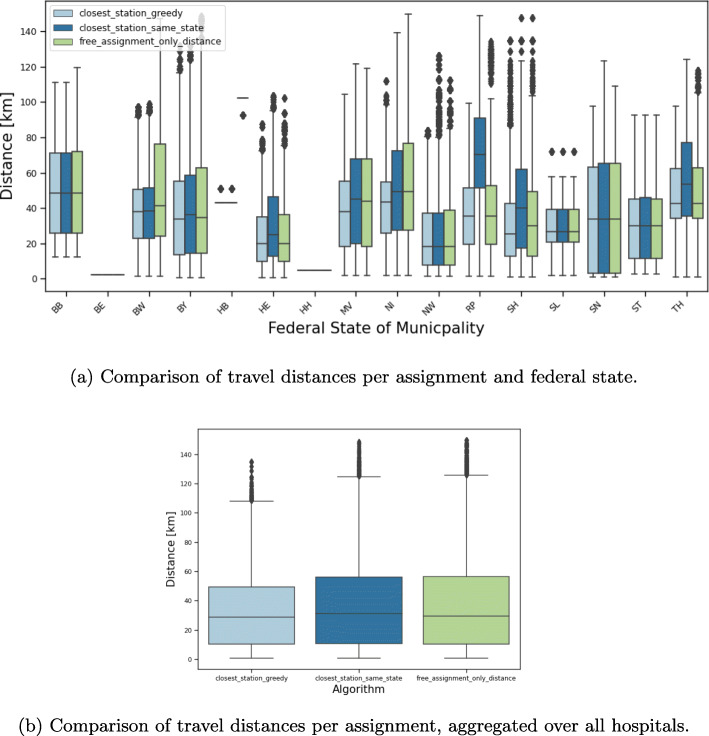
Distribution of travel distances (as linear distances in kilometers) for assignments i, ii, and iii in the university hospital scenario

As university hospitals are often located in large cities, many patients reach the vaccination facility within an acceptable distance. But especially patients from rural areas have to travel a considerable distance to their facility. While Fig. 6 shows the median distance of each municipality on a map, for an informed decision, it is also of interest to see the range of distances scaled by the population size. The maximum distance between a municipality center and the closest university hospital is 135 km (affecting only few people), while the median for this assignment is 30 km. Figure 8 shows boxplots of the distances for assignment i compared to assignment ii and iii aggregated over the total German population (Fig. 8b) and grouped by states (Fig. 8a). Clearly, additional constraints such as vaccination in the own federal state or capacity restrictions increase the distances travelled by the patients.

When the university hospital solution is being chosen, each facility will face a median of around 12 000 patients per week, with 80% of the facilities seeing around 6 000 to 24 000 (22 000 for assignment i) patients per week.

#### Greenfield planning (IV)

The general results for this scenario are given in Table 6.

**Table 6:** Results for Greenfield scenario

	Strategy	Med. Distance	#Locations	#Physicians	Med. Utilisation
	*free_assignment_15km*	8.6 km	363	2091	86%
	*free_assignment_30km*	12.8 km	220	2002	100%
	*free_assignment_50km*	25.8 km	81	2000	100%
	*free_assignment_75km*	34.9 km	39	2000	100%

**Fig. 9:**
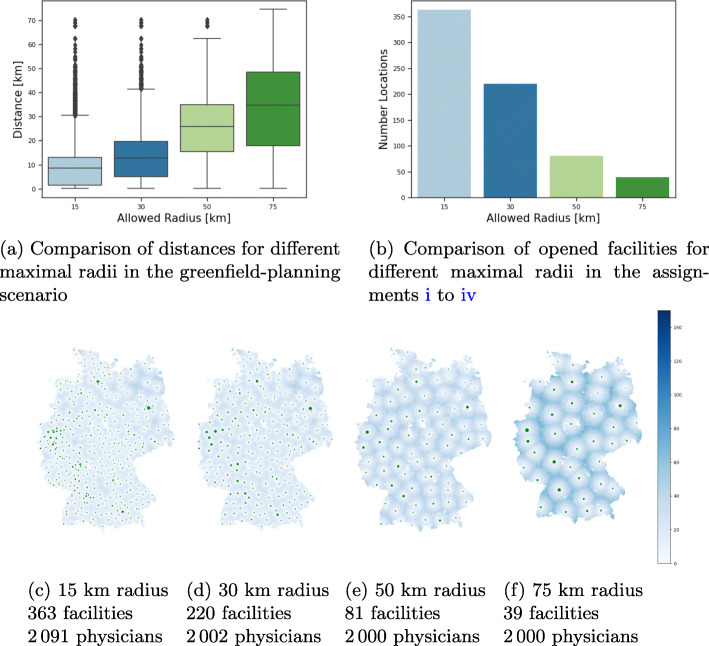
Comparison of free assignments in the greenfield planning scenario with respect to different maximal radii. Note that municipalities whose closest facility exceeds the defined radius are being assigned to their closest facility. Therefore larger radii than allowed can occur. The marker sizes are proportional to the number of assigned patients

Figures 9c–f clearly show that an even distribution of vaccination centres across Germany is optimal for minimizing distances. With an increase of the allowed travel distance, the number of required vaccination facilities decreases significantly, which can be seen in Fig. 9b. In some federal states, the density of health departments is rather sparse, e.g. in Mecklenburg-Vorpommern (in the north-east of Germany, see Fig. 3a). Hence, the allowed radii of 15, 30, and 50 km can not be met for all municipalities. In Fig. 9c and d this is reflected in darker areas mostly in the North-East. These regions are also reflected in the outliers of the boxplot in Fig. 9a, where the distances of all German patients are visualized.

All four assignments require up to 2 091 physicians. Due to the complexity of the model, these free assignments have not been computed up to optimality. Therefore, we cannot clearly say whether the increase in doctors for smaller radii stems from unbalanced population densities on a smaller scale or simply from unoptimal assignments.

For the sake of finding a compromise, it might however be more reasonable to not look only on maximal distances, but certain quantiles, such as the median or the 75% quantile of resulting distances, which can also be found in Fig. 9a. For example, the 50 km scenario iii has a median distance of 25 km, i.e. 50% of all patients have a one-way journey length of 25 km or less. Looking at the 75% quantile, the distance rises to around 35 km.

#### Comparison

**Fig. 10:**
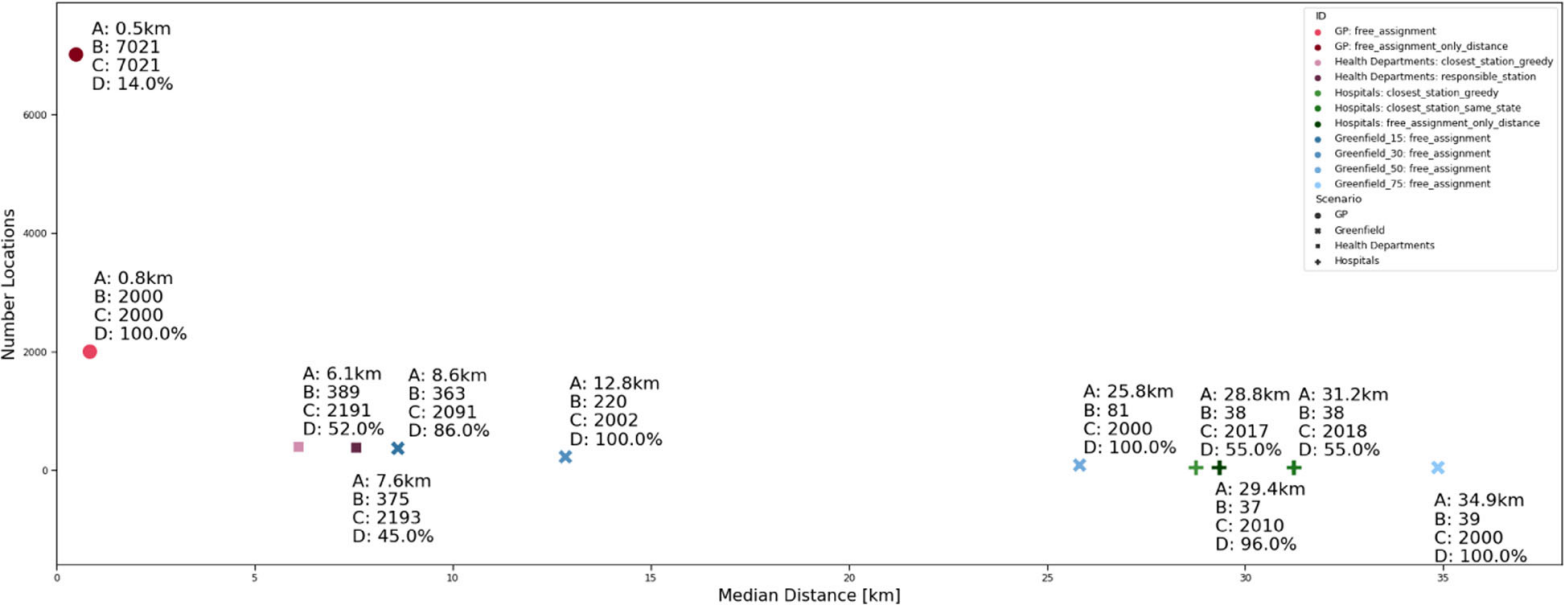
Overview of median airline distances versus the number of required vaccination locations for all considered scenarios. The labels depict the detailed values of the median distance (A) / the number of locations (B) / the required number of doctors (C) / the median utilisation rates (D)

In comparison, one can see a clear trade-off between the travel distances and the required number of vaccination facilities. For the median distance, this curve is depicted in Fig. 10 for all considered scenarios. It also shows the differences in required physicians and the resulting utilisation rates. However, as stated earlier, the *free_assignment* solutions have not necessarily been solved to optimality due to the complexity of the model.

**Fig. 11:**
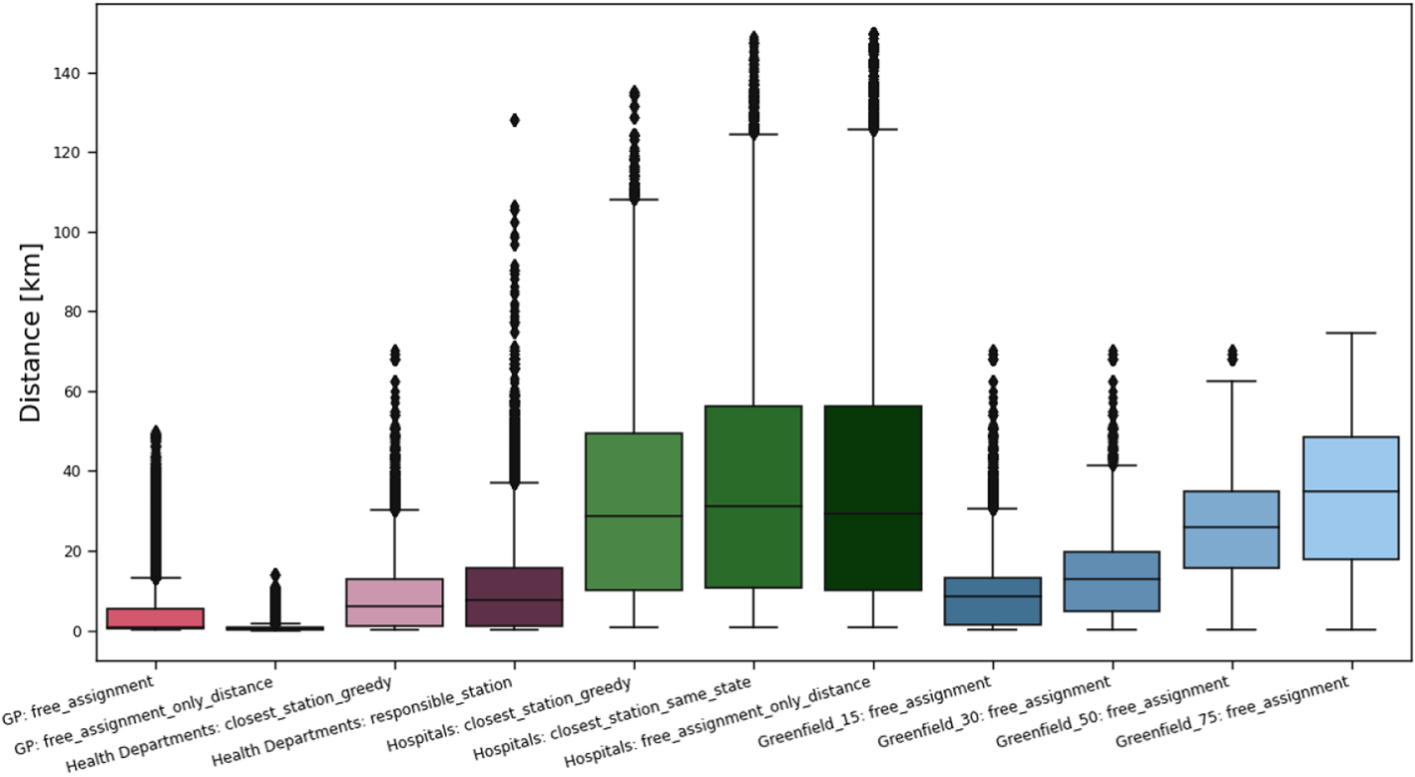
Overview of airline distance distributions for all considered scenarios

Since the median of the distances does not show the whole picture, in Fig. 11, the distance distributions are compared for all scenarios. For example, the travel journeys in the *University Hospital* scenarios are similar in the median to the *Greenfield* scenario with an allowed radius of 75 km, where the number of locations is comparable. However, 25% of the population face one-way travel journeys of 60 - 145 km even to their closest university hospital, while the distances in the *Greenfield* scenario are bounded by 75 km. While the median of the shortest airline travel distances is very acceptable (<10 km) for the health department scenarios and hence comparable to the *general practices* scenario, for around 1% of the population, due to the disadvantageous location of some health departments, the maximal travel journey can be 30 km up to 70 km (128 km).

**Fig. 12:**
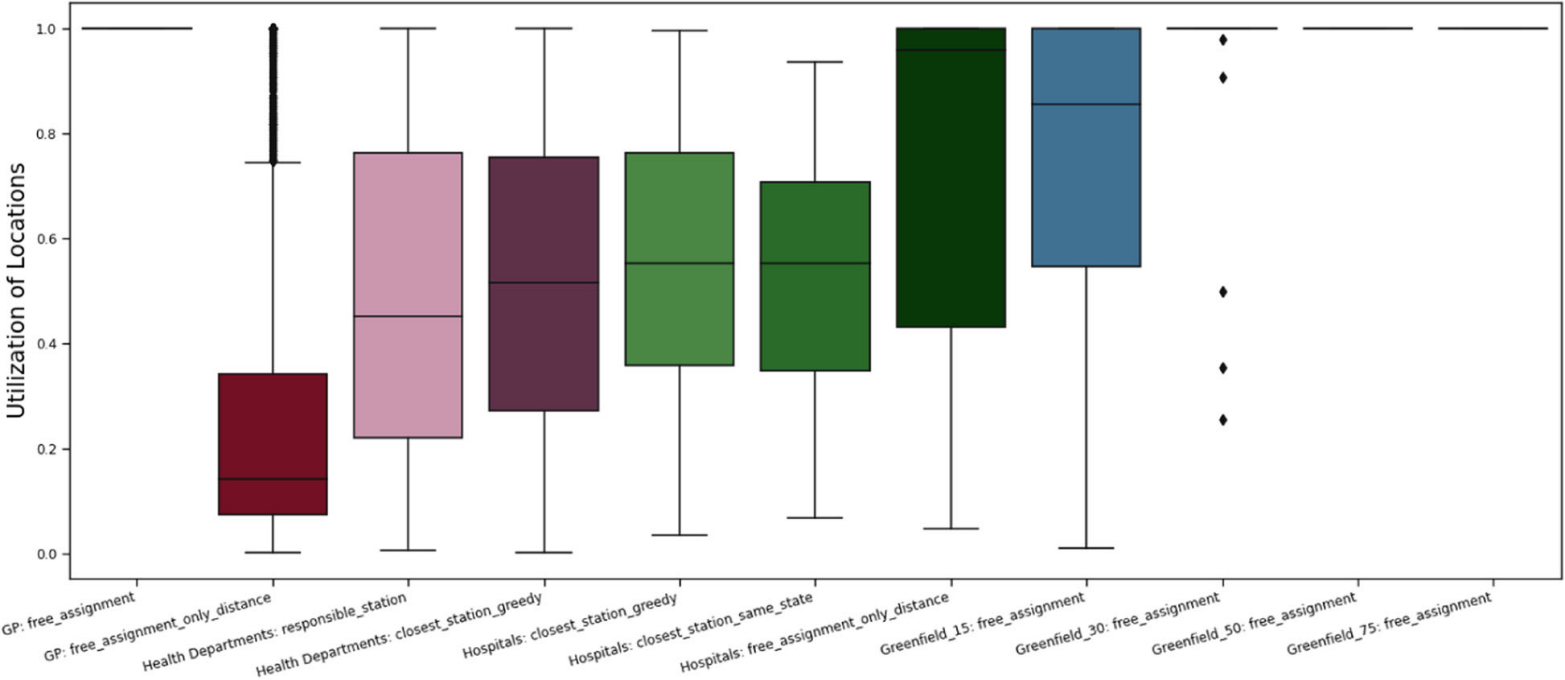
Overview of the distribution of the vaccination centres’ utilisation rates for all considered scenarios

In Fig. 12, the induced physicians’ utilization rates of the individual scenarios are illustrated. Again, some *free assignments* need to be considered with caution, since an optimal solution may distribute patients even further, leading to then higher utilization rates. The main result is, that the *general practices* scenario with around 7 000 open locations has the worst utilization rates by far. This may not be a problem when the vaccination process has become established and the vaccines are distributed in individual packages. However, if the vaccines are only shipped in larger containers due to complex transport requirements, this scenario can only be implemented meaningfully once the vaccine availability is very high. Also, the scenarios that are defined by administrative assignment rules have worse utilization rates on average than the *free assignments*.

When no administrative or technical requirements prespecify assignments, the *free assignments* are understandably advantageous, as more people can be covered within acceptable distances. This can be achieved while maximizing the physicians’ utility rates. Depending on how one weights the establishment and fix costs of a vaccination centre against the accessibility of vaccinees, a *Greenfield* scenario with 15 to 30 km allowed radius might be the best trade-offs in this case. However, due to the distribution of vaccines among the states, administrative bounds may be of concern. For example, in the *university hospital scenario*, there are federal states that do not have any such facility. Assignments with or without crossing state borders may therefore lead to discussions about intergovernmental agreements. Also, the organization of vaccination registration may be easier and more comprehensible, if clear responsibilities can be communicated.

### Sensitivity analysis

Beyond the presented results, we have also investigated the sensitivity of the assignments with respect to several uncertain parameters. These are the composition of the population, the capacity per doctor and the total available amount of vaccine. However, all prove to have little effect in our static approach. We tested to consider only the population of persons being 65 or older. Although the proportion of seniors varies widely among German municipalities [29], our results did not show a significant variation in the structure of solutions and the performance indices of a nationwide level. However, since municipalities with a higher proportion of elderly people are typically more rural and rural areas are usually more distant from the vaccination facilities, in some states there is a shift towards an increased median travel distance and more required doctors.

The variation of available doses or the variation of the capacity of one physician have in principle only linear effects in all scenarios that do not consider bounds on the vaccination facility capacity. That is, the number of required physicians roughly doubles if the number of available vaccines doubles. However, since some municipalities are very small, saturation effects on some municipalities can occur with an increased capacity. When respecting absolute capacities, an increasing number of patients obviously will change the assignment or even render it invalid when reaching the bounds. However, due to a lack of suitable data, we only considered bounds relative to the outbound patient size and by scaling these to the capacities, the solution does not alter in structure, but basically only scales the assigned patients accordingly. If data about absolute bounds is provided, it would be possible to consider this.

For the free assignments (greenfield planning), where patients are assigned to a suitable vaccination center within a prespecified radius and not all possible vaccination centers have to open, this radius of course has a huge impact on both the median travel distance as well as the location of the selected facilities. When planning the facility locations, this should be taken into account, since the COSMO Study [10] evaluated a significant drop of vaccination willingness in case the travel time (outward and return journey) exceeds 1 hour. With this in mind, our results should also motivate politicians to enable vaccination processes that are not restricted by administrative borders, since these automatically lead to avoidable peaks in travel journeys for people from border regions.

## Outlook

The situation of planning a population-wide vaccination campaign against a deadly virus several months in advance of the approval of any vaccine in the middle of a pandemic is unique in history. It is therefore not surprising that the assumptions and knowledge of many parameters regarding the availability and shelf life of vaccine doses and the technology required are constantly changing. The present study can thus only reflect the current state of discussion. It is also only suitable to decide on the question of the type of vaccination locations and gives a rough allocation of citizens to places.

The tactical planning gives rise to new questions. Instead of optimizing the conflicting objectives lexicographically, it could be beneficial to analyse them in a multi-objective optimization approach, in order to highlight good compromises that, e.g., increase the distances only for very few people while balancing the required number of doctors at the individual facilities. Another aspect that could be taken into account is the heterogeneous willingness in the population to be vaccinated, which on the one hand possibly depends on the local occurrence of infection, but on the other hand also on the distance to the vaccination site.

Evaluating the vaccination assignment as a time-dynamic organizational problem may also include varying vaccine availability over time, changing demand in the population and a potential need of revaccinating patients after a certain number of weeks or months.

The operational planning of such a vaccination campaign must then be carried out at the state level. This allows also for taking regional characteristics and actual road distances into account. It should also be stated that a more refined location planning would also compensate for the flaw of very different definitions of a municipality throughout Germany in terms of area and population size.

## Data Availability

Input data (such as population data) can be found online at the referenced locations. Solution data can be made available upon request to people with legitimate interest. Any request for data sharing may be sent to the corresponding author at her email address neele.leithaeuser@itwm.fraunhofer.de.
